# Fully Guided Implant Placement and Immediate Provisionalization in the Maxillary Aesthetic Zone Using Prefabricated Restorations

**DOI:** 10.1155/crid/8990886

**Published:** 2025-09-15

**Authors:** Vincent J. J. Donker, Henny J. A. Meijer, Arjan Vissink, Gerry M. Raghoebar

**Affiliations:** ^1^Department of Oral and Maxillofacial Surgery, University of Groningen, University Medical Center Groningen, Groningen, the Netherlands; ^2^Department of Restorative Dentistry, University of Groningen, University Medical Center Groningen, Groningen, the Netherlands

**Keywords:** alveolar ridge preservation, case report, delayed implant placement, digital workflow, prefabricated restoration

## Abstract

**Introduction:** When replacing a single tooth in the aesthetic zone with an implant, alveolar ridge preservation is necessary in cases with extensive buccal bone defects. Prosthetically driven implant placement in the preserved ridge, followed by an immediate patient-specific temporary restoration, can be achieved with digital treatment planning. A digital workflow incorporates intraoral optical scanning and cone beam computed tomography, enabling a three-dimensional clinical and radiographic anatomy assessment. Furthermore, it facilitates fully guided implant placement by means of computer-assisted surgery and the prefabrication of temporary restorations with a CAD/CAM titanium abutment.

**Materials and Methods:** Three patients with a failing tooth in the maxillary aesthetic zone and a buccal bone defect > 5 mm underwent alveolar ridge preservation. Four months later, a digital workflow was used to place the implant, which was restored with a prefabricated temporary restoration with a patient-specific titanium abutment. The definitive restoration was placed 3 months later. Clinical, aesthetic, radiographic, and patient-reported outcomes were assessed before treatment, 6 weeks after temporary restoration, and 1 month and 1 year following definitive restoration.

**Results:** In all three cases, wound healing after alveolar ridge preservation was uneventful, and the implants could be placed and restored with a temporary restoration, as planned. At the final follow-up, healthy peri-implant tissues were observed with good aesthetics and high patient satisfaction.

**Conclusion:** The three reported cases demonstrate the potential of a digital workflow for delayed implant placement with provisionalization using prefabricated restorations in preserved ridges within the maxillary aesthetic zone.

## 1. Introduction

In the maxillary aesthetic zone, failing single teeth are typically replaced by immediate implants [1, 2]. However, large bone defects and a lack of primary stability, significant mucosal recession, or extensive infection may render the site unsuitable for immediate implant placement [3, 4]. In these cases, extracting the tooth and allowing spontaneous healing of the alveolus lead to progressive physiological resorption of buccal bundle bone, since the blood supply from the periodontal ligament is lost [5–7]. The extent of resorption varies depending on the initial buccal bone thickness and the surgical technique employed during tooth extraction [8, 9]. Various interventions aimed at limiting the extent of alveolar ridge alteration and at facilitating delayed implant placement have been proposed, mainly involving grafting the alveolar socket with autologous bone or biomaterials [10, 11]. Especially within the aesthetic zone, where bone is critical for soft tissue support, alveolar ridge preservation has a major impact on the outcome of implant-supported restorations.

After alveolar ridge preservation, implants must be placed in the correct three-dimensional position [12]. Moreover, the soft tissues need contouring to get a natural emergence profile, which can be achieved by using a temporary restoration [13, 14]. Digital treatment planning enhances predictability by facilitating implant positioning and restoration design. Such a digital workflow incorporates intraoral optical scanning and cone beam computed tomography (CBCT), enabling a three-dimensional setup to assess the clinical and radiographic anatomy and the subsequent prosthetically driven implant planning [15, 16]. The planned implant position can be transferred to the patient using static computer-assisted implant surgery with a surgical template. Furthermore, a digital workflow enables prefabrication of a patient-specific temporary restoration, which can be delivered immediately after implant placement.

Several techniques for direct chairside implant restoration have been described, ranging from freehand resin composite modeling and prefabricated shells relined on a temporary abutment to fully personalized computer-aided design and manufacturing (CAD/CAM) restorations [17, 18]. The choice of restoration technique depends on the level of surgical guidance and the resulting deviation from the planned implant position [19, 20]. Furthermore, there are a variety of abutment options to choose from. The submucosal part should consist of a biocompatible material, with titanium being preferred over polyether ether ketone (PEEK) or resin composite from a histological perspective [21]. Apart from stock abutments, patient-specific CAD/CAM abutments are increasingly being used and have shown excellent clinical behavior [22]. However, reports on their use for immediate provisionalization in the maxillary aesthetic zone are limited.

The objective of this case report is to describe a digital workflow for fully guided implant placement, with immediate provisionalization, using prefabricated restorations with patient-specific titanium abutments, in preserved ridges of the maxillary aesthetic zone. Additionally, it reports the clinical, radiographic, and patient-reported outcome of three cases, including the 1-year follow-up after placing the definitive restoration.

## 2. Study Design

Three patients, who underwent single-tooth restorations supported by implants placed in preserved ridges within the maxillary aesthetic zone, were followed for 1 year after the definitive restoration. Recruitment of patients, treatment, and follow-up took place in the Department of Oral and Maxillofacial Surgery at the University Medical Center Groningen (UMCG), the Netherlands. The research protocol was reviewed by the Medical Ethics Review Board of the UMCG (METc 2021/430). This manuscript was written following the CARE guidelines for case reports [23].

## 3. Patient Information and Clinical Findings

Initial eligibility criteria included being at least 18 years of age at the time of treatment and patients in need of a single implant-supported restoration in the maxillary aesthetic zone (from the first premolar to the first premolar). After tooth extraction, the osseous defect was evaluated using bone sounding at the midfacial, mesial, and distal aspects with a periodontal probe. If the buccal socket wall defect was greater than one-third of the mesiodistal width between adjacent teeth and the vertical depth of the defect exceeded 5 mm, the patient could be included. Additional criteria included adequate anatomical conditions, sufficient mesial–distal, buccal–lingual, and interocclusal space to support a well-shaped restoration, good oral hygiene (plaque score below 20%), no medical or systemic contraindications for surgery, a nonsmoking status, absence of severe bruxism or parafunctional habits, and no active, untreated periodontitis or infections at the planned implant site or surrounding tissues. Patients with a history of oro-maxillofacial radiotherapy were excluded.

The three consecutively recruited patients agreed to participate in the study (Table 1). An explanation of the costs, benefits, and risks of an implant-supported restoration, along with possible alternative treatment options, was provided. Written informed consent was obtained from the participants before enrollment.

## 4. Timeline

The patients underwent alveolar ridge preservation with a 4-month healing period, followed by fully guided delayed implant placement and immediate provisionalization and, 3 months later, definitive restoration placement, as outlined in Table 2. One case is presented for illustrative purposes.

## 5. Diagnostic, Laboratory, and Bone Augmentation Procedures

**Figure 1:**
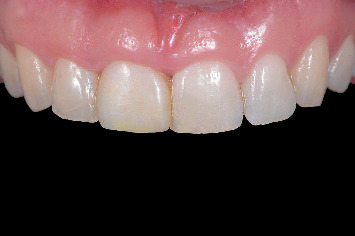
Clinical view of a patient with endodontic failure of the maxillary right central incisor.

**Figure 2:**
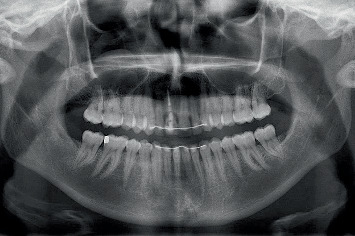
Radiographic view showing the failing maxillary right central incisor and donor site.

Before the treatment, digital photographs (Nikon D750, Nikon Corporation, Tokyo, Japan) and intraoral optical scans (TRIOS 3, 3Shape, Copenhagen, Denmark) were made of the maxillary and mandibular arches and occlusion (Figure 1) and sent to the dental laboratory to fabricate an acrylic removable partial denture to replace the failing tooth. In addition, an orthopantomogram (ProMax 3D Max ProFace, Planmeca, Helsinki, Finland) was made to assess the bone augmentation donor site (Figure 2) for alveolar ridge preservation.

**Figure 3:**
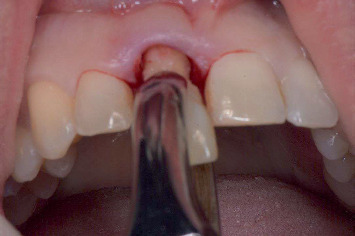
Minimally traumatic extraction of the failing tooth.

**Figure 4:**
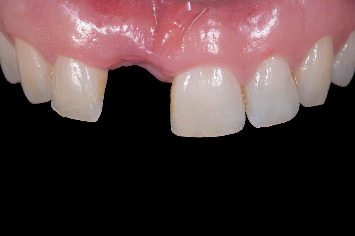
Clinical view after tooth extraction and alveolar ridge preservation.

The patients began using a 0.12% chlorhexidine mouthwash twice daily and an antibiotic regimen (amoxicillin 500 mg t.i.d. or clindamycin 300 mg q.i.d. for penicillin-allergic patients) starting the day before surgery and continued for 7 days postoperatively. Local anesthesia (Ultracain D-S forte, Sanofi-Aventis Deutschland GmbH, Frankfurt am Main, Germany) was administered, and the failing tooth was extracted as minimally traumatically as possible using an intrasulcular incision, periotome, and forceps (Figure 3). Any remaining ligament fibers and granulation tissue were removed with a bone curette and sterile gauze. Bone grafts were harvested from the maxillary tuberosity, and an oval-shaped full-thickness mucosal graft was obtained from the same region. The donor site was sutured with 4-0 Vicryl (Ethicon, Johnson & Johnson, New Brunswick, New Jersey, United States). Next, 3–4 mm of the periosteum was carefully reflected on the buccal side of the socket, and the bone graft was inserted with the cortical side facing outwards. Any remaining autogenous bone particles were mixed with bovine bone granules (Geistlich Bio-Oss, Geistlich Pharma AG, Wolhusen, Switzerland) and packed into the socket. The site was sealed with a mucosal graft and sutured using 4-0 Ethilon (Johnson & Johnson). The alveolar ridge preservation protocol published by Raghoebar et al. [24] was followed by monitoring wound healing and removing sutures 2 weeks postoperatively (Figure 4). All the surgical procedures were performed by one experienced oral and maxillofacial surgeon (G.M.R.). During the healing phase, the patients used a removable partial denture.

## 6. Diagnostic, Planning, and Implant Placement Procedures

**Figure 5:**
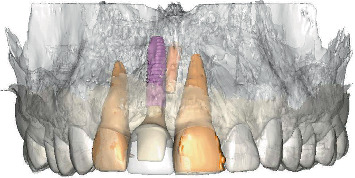
Prosthetically driven implant planning in the implant planning software.

**Figure 6:**
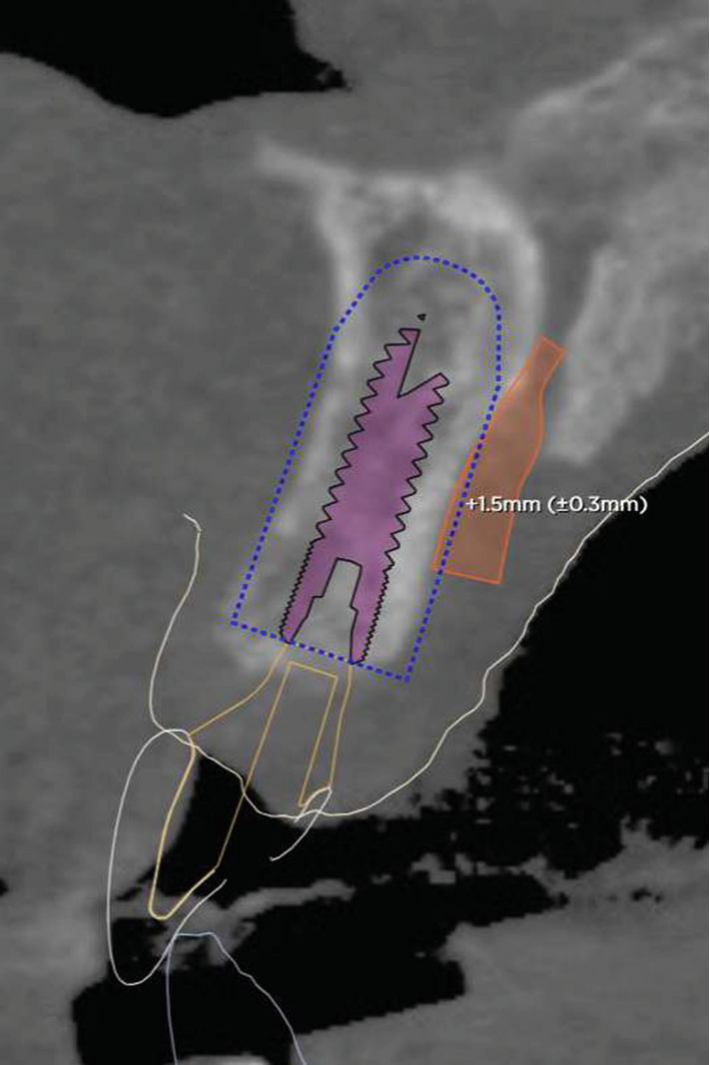
Sagittal radiographic view of implant positioning.

**Figure 7:**
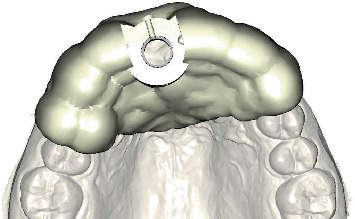
Computer-aided design of the surgical template, occlusal view.

**Figure 8:**
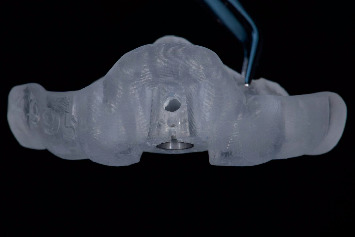
Manufactured surgical template based on the digital treatment plan.

After a 4-month healing period, intraoral optical scans were made, as well as a CBCT scan (ProMax 3D ProFace) for three-dimensional clinical and radiographic assessment of the preserved ridge. This data was sent to an implant planning service (Azento, Dentsply Sirona), where a technician created a digital setup (Figure 5) to facilitate prosthetically driven implant planning (Figure 6) with implant planning software (Simplant Pro 18.5, Dentsply Sirona, Hasselt, Belgium). After the treatment plan was approved by the surgeon and prosthodontist, a patient-specific gold-shaded titanium abutment and polymethyl methacrylate (PMMA) crown (Atlantis CustomBase Solution, Dentsply Sirona), as well as a fully guided surgical template, were manufactured (Figures 7 and 8).

**Figure 9:**
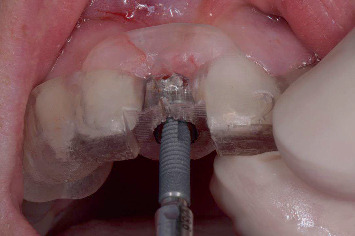
Fully guided implant placement in the preserved ridge.

The day before implant placement, the patients began oral disinfection with a 0.12% chlorhexidine mouthwash twice a day and had to continue for 7 days. In addition, they took a prophylactic antibiotic (amoxicillin 2 g or clindamycin 600 mg in case of penicillin allergy) 1 h before the treatment. The surgical procedure was performed using local anesthesia (Ultracain D-S forte). Using the fully guided surgical template, a soft tissue punch was made, followed by a flapless guided osteotomy in the preserved ridge and placement of a straight bone-level implant with a tapered apex and conical connection (Astra Tech Implant EV, Dentsply Sirona), following the manufacturer's instructions (Figure 9).

## 7. Restorative Procedures

**Figure 10:**
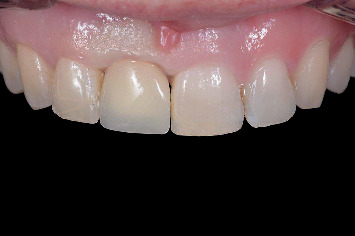
Clinical view immediately after implant placement and provisionalization with the prefabricated restoration.

**Figure 11:**
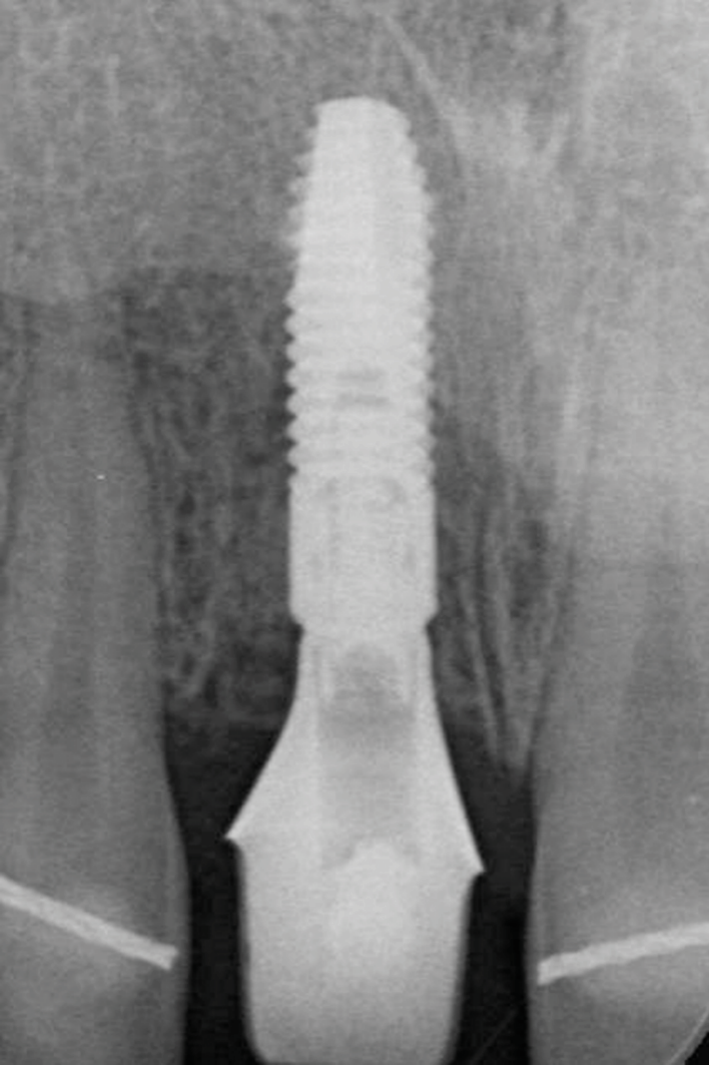
Radiographic view of implant in preserved ridge and temporary restoration.

The implants were immediately restored chairside with the prefabricated temporary restoration without requiring any modification. The PMMA crown was attached to the patient-specific CAD/CAM abutment using light-polymerizing resin cement (Panavia V5, Kuraray, Tokyo, Japan) and secured onto the implant with a torque of 15 Ncm using a manual torque controller (Torque Wrench EV, Dentsply Sirona). The screw access hole was sealed with polytetrafluoroethylene (PTFE) tape and resin composite (Figures 10 and 11). The temporary restoration was designed in such a way as to avoid centric and eccentric contact, and the patients were advised to avoid excessive force during the osseointegration period, and were instructed on oral hygiene.

**Figure 12:**
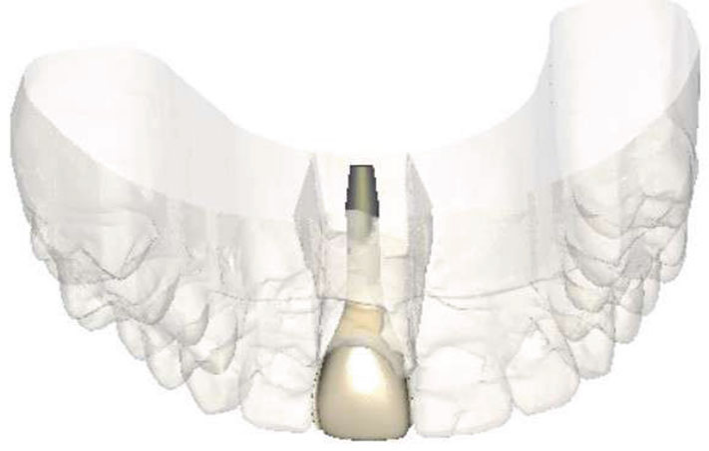
Computer-aided design of the definitive patient-specific abutment and crown.

**Figure 13:**
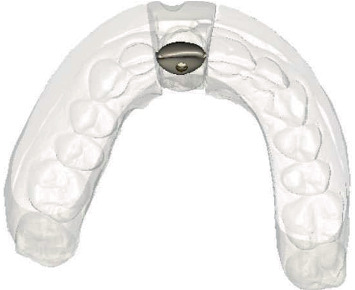
Computer-aided design of the definitive crown with facial cutback, occlusal view.

**Figure 14:**
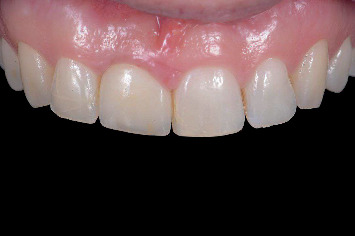
Clinical view of the definitive restoration at the final follow-up appointment.

**Figure 15:**
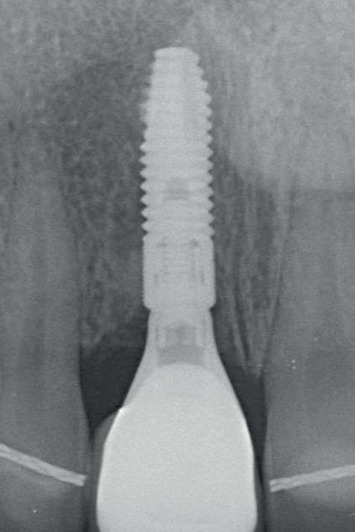
Radiographic view of the implant and definitive restoration at the final follow-up appointment.

Following the 3-month osseointegration period, the soft tissue emergence profile was scanned immediately after removing the temporary restoration. A scanbody (Atlantis IO FLO, Dentsply Sirona) was used to capture the implant position. The files were sent to a centralized production facility (Atlantis, Dentsply Sirona) where a screw-retained restoration, comprising a zirconia (Cercon xt ML, Dentsply Sirona, Charlotte, North Carolina, United States) crown with facial cut-back and a patient-specific gold-shaded titanium abutment (Atlantis CustomBase Solution), was designed and manufactured (Figures 12 and 13). These components were then sent to a local dental laboratory where the restoration was finished with a buccal porcelain veneer. The patients were present during this process to ensure the restoration could be fitted and evaluated for color. The definitive restoration was secured onto the implant with a torque of 25 Ncm, and the screw access hole was sealed with PTFE tape and resin composite (Figures 14 and 15). Oral hygiene instructions were provided again. All the restorative procedures were performed by two prosthodontists (H.J.A.M., V.J.J.D.).

## 8. Follow-Up and Case Outcomes

This report follows the Core Outcome Sets and Measurements for Bone Augmentation (BA-COSM) and Implant Dentistry (ID-COSM) by [25], consisting of clinical, aesthetic, radiographic, and patient-reported outcomes. The assessments were done before the treatment (Tpre), 2 weeks after alveolar ridge preservation, directly following placement of the implant with a temporary restoration (T0), 6 weeks after healing with the temporary restoration in situ (Ttemp), and then 1 month (T1) and 1 year (T12) after definitive restoration placement (Figures 14 and 15).

The mean buccal bone defect after the tooth was extracted measured 9.7 mm (range: 8–12 mm). In all three cases, the donor site for the bone graft was the maxillary tuberosity. After the alveolar ridge preservation procedure, wound healing was uneventful and none of the patients presented with edema or infection. All the implants could be placed in the planned position with adequate stability and without the need for additional bone augmentation.

In the three cases described, all the implants could be restored immediately with the prefabricated temporary restoration. The temporary restorations were in situ and complication-free until after the osseointegration period, but the PMMA crowns debonded from the CAD/CAM titanium abutment in all cases during removal for the intraoral optical scanning for the definitive restoration and required recementation. At T12, no implants or definitive restorations had been lost and survived complication-free, resulting in a 100% survival and success rate for these three cases according to the modified US Public Health Service (USPHS) criteria for evaluating implant-supported restorations [26, 27].

The clinical outcomes consisted of the modified plaque index [28], the modified sulcus bleeding index [28], the gingival index [29], buccal keratinized mucosal width [30], and probing pocket depth at four sites (mesial, distal, buccal, and palatal). The aesthetic outcomes were assessed from intraoral photographs (Nikon D750) made at all timepoints and encompassed the papilla index [31], change in midfacial mucosal level (MML), and the modified pink esthetic score and white esthetic score (PES/WES) [32]. The radiographic assessment included change in marginal bone level (MBL), measured between the timepoints on calibrated intraoral radiographs using dedicated software (DicomWorks, Biomedical Engineering, UMCG, the Netherlands). A questionnaire with a visual analogue scale was used to assess the patient-reported outcome measure (PROM). All the data were collected by one trained observer. The mean values and standard deviations were calculated using statistical software (IBM SPSS Statistics, Version 28.0, Armonk, New York, United States). The outcomes of all three cases are shown in Table 3.

## 9. Discussion

This case report describes a digital workflow for fully guided implant placement with immediate provisionalization, utilizing restorations with patient-specific titanium abutments in preserved ridges in the maxillary aesthetic zone. All the implants could be placed and restored as planned, without the need for any adjustments.

The clinical, aesthetic, radiographic, and patient-reported outcomes are similar to those of a previous study by the same clinic, which involved immediate provisionalization of implants placed freehand in preserved ridges using a conventional workflow [33]. Notably, the digital workflow offers several distinctions and advantages in the planning and execution of implant treatment following alveolar ridge preservation compared to conventional methods, enabling these outcomes to be achieved with greater predictability for both the surgeon and the prosthodontist.

The utilization of an implant planning software facilitates the integration of intraoral optical scans and CBCT scans, thereby enabling a comprehensive three-dimensional evaluation of the clinical and radiographic anatomy during the diagnosis and planning. This integration not only supports a prosthetically driven implant position but also allows for a priori assessment of the available bone volume within the preserved ridge. Consequently, it is possible to determine in advance whether additional buccal contour augmentation is required to ensure a minimum bone wall thickness of 2 mm buccal to the implant [12].

Fully guided static computer-assisted implant surgery results in more accurate implant placement compared to conventional freehand techniques, thereby allowing the placement of a prefabricated restoration. In the present study, all the implants were successfully positioned, as planned, using a fully guided surgical template, and the prefabricated restorations were fitted without adjustment, demonstrating accurate translation of the digital planning into clinical execution. Nonetheless, a fully guided surgical protocol can still result in deviations between planned and actual implant positions. Such deviations typically originate during the initial drilling phase and persist throughout the remainder of the guided osteotomy procedure. While some studies report these deviations as being clinically imperceptible (95% CI: 2.06°–5.38°), they may still cause a misfit of the prefabricated temporary restorations [34, 35]. While this was not observed in the present study, a limitation is that implant deviation was not analyzed and the sample size was small. Further studies with larger cohorts are needed to investigate whether significant deviations occur after implant placement and whether these are clinically relevant for the employed digital workflow.

In the aforementioned study employing a conventional workflow, an impression was required following implant placement to facilitate the design and fabrication of a customized temporary restoration in the dental laboratory [33]. This approach also necessitated an additional clinical visit for temporary restoration placement. In the present study, the digital workflow eliminated the need for an impression after implant placement and an additional appointment, because of the use of prefabricated restorations. This approach enhanced patient comfort and reduced chair time.

Additional advantages of a digital workflow for implant restoration include reduced procedure time and patient discomfort, as intraoral optical scanning is less invasive than conventional impression techniques [36, 37]. Furthermore, CAD/CAM can decrease both production time and fabrication costs. However, it should be noted that fabricating the buccal veneer on the crown in the present study extended the overall production time [38, 39].

In conclusion, the outlined planning, surgical, and restorative considerations offer compelling reasons to choose a digital workflow over conventional methods to achieve favorable outcomes for implants placed in preserved ridges within the maxillary aesthetic zone.

## Figures and Tables

**Table 1:** Patient information.

	**Case 1**	**Case 2**	**Case 3**
Sex	Female	Female	Male
Age in years	28	30	50
Reason for treatment	Root resorption	Endodontic failure	Crown fracture
Implant position	Maxillary right central incisor	Maxillary right central incisor	Maxillary right central incisor
Implant diameter	3.6 mm	4.2 mm	4.2 mm
Implant length	13 mm	13 mm	11 mm

**Table 2:** Protocol of the alveolar ridge preservation, followed by fully guided delayed implant placement and immediate temporary restoration, with definitive restoration placement after osseointegration.

**Treatment phase**	**Procedures**
Diagnostic	Intraoral optical scans
	Orthopantomogram

Laboratory	Fabrication of a removable partial denture

Bone augmentation	Tooth extraction
	Bone graft harvesting
	Bone graft placement

Diagnostic	Intraoral optical scans
	Cone beam computed tomography scan

Planning	Digital setup
	Prosthetically driven implant planning
	Computer-aided design and manufacturing of a titanium abutment, temporary restoration and surgical template

Implant placement and temporary restoration	Flapless fully guided static computer-assisted implant surgery
	Prefabricated temporary restoration placement

Definitive restoration	Intraoral optical scans with a scanbody
	Computer-aided design and manufacturing of a definitive abutment and crown
	Porcelain veneering of the crown
	Definitive restoration placement

**Table 3:** Outcomes: Modified plaque index, modified sulcus bleeding index, gingival index (range 0–3), buccal keratinized mucosal width, and pocket probing depth at T12. Papilla index (range 0–4), midfacial mucosal level change from Tpre to T12. Modified pink esthetic score and white esthetic score (range 0–10). Marginal bone level change from T0 to T12. Patient-reported satisfaction (range 1–10).

	**Case 1**	**Case 2**	**Case 3**	**Mean (SD)**
Modified plaque index	Score 0	Score 0	Score 0	
Modified sulcus bleeding index	Score 0	Score 0	Score 0	
Gingival index	Score 0	Score 0	Score 0	
Keratinized mucosal width (mm)	≥ 2	≥ 2	≥ 2	
Pocket probing depth (mm)				
Mesial	2	3	4	3 (1)
Distal	2	2	2	2 (0)
Buccal	3	3	3	3 (0)
Palatal	2	2	2	2 (0)
Papilla index				
Tpre mesial	Score 1	Score 3	Score 3	
Tpre distal	Score 1	Score 2	Score 2	
T12 mesial	Score 3	Score 3	Score 2	
T12 distal	Score 2	Score 2	Score 2	
Midfacial mucosal level change (mm)^a^	0.0	0.4	−0.2	0.1 (0.3)
Pink esthetic score				
Tpre	7	8	8	7.7 (0.6)
Ttemp	6	7	6	6.3 (0.6)
T12	8	7	8	7.6 (0.6)
White esthetic score				
Tpre	8	9	7	8.0 (1.0)
Ttemp	7	7	7	7.0 (0.0)
T12	9	9	7	8.3 (1.2)
Marginal bone level change (mm)^a^	−0.10	−0.11	0.00	−0.07 (0.06)
Patient-reported satisfaction				
Tpre	7	8	2	5.7 (3.2)
Ttemp	8	8	8	8.0 (0.0)
T12	9	9	8	8.7 (0.6)

Abbreviations: T1, 1 month after definitive restoration; T2, 1 year after definitive restoration; Tpre, before treatment; Ttemp, 6 weeks after temporary restoration.

^a^A negative value indicates soft tissue recession/marginal bone loss.

## Data Availability

The authors confirm that the data supporting the findings of this study are available within the article.
